# The Immediate Cure of Whooping Cough

**Published:** 1887-04

**Authors:** 


THE IMMEDIATE CURE OF WHOOPING COUGH.—Dr. Mohn of Christiana communicates to his Norwegian confreres a new method of treatment for whooping cough, for which he claims remarkable results, the disease being cured in a single night. His plan consists simply in the thorough disinfection, by means of burning sulphur, of the rooms, clothing, etc., used by the affected children. The children are taken out of the room, the bedding, furniture and play-things are exposed and two ounces of sulphur are burned for every 100 cubic feet of space in the room. After the room has been thus exposed to the sulphurous acid fumes the affected children are allowed to return and occupy it. As a result of this treatment it is claimed that attacks of coughing are immediately alleviated, and often entirely disappear. —*Revue des Sc. Med.*

---

DR. B. C. WINDLE of Dublin, in a paper read before the British Dental Association on "Man's Lost Incisors," reaches the following conclusions:

1. Man's original dentition included six incisors.
2. Man's lost incisor in the lateral In3.
3. This loss is consequent upon the contraction of the anterior part of the jaw.
4. Suppression of the two present lateral incisors is now taking place.
5. Conical teeth are a reversion to the primitive type.

---

HENNIG and Rauber report in *Virchows Archives* the case of a young dairy maid who gave birth to a child with a tail. This caudal appendage was 27 m. m. (1½ inch) in length, contained two vertebræ and had hairs on its extremity. The authors emphasize the importance of moral impression from association with cows in this case.

---

ST. LOUIS has been lately blessed by the arrival of a Chinese doctor, who offers to break his own arm if another surgeon will do the same, and then prove the superiority of his treatment over that of the other.—*Medical Review.*

---

PROF. F. MAGNI, the distinguished Italian ophthalmologist, died Feb. 2, 1887.

THE following physicians were appointed at Feb. 8th meeting of the French Academy of Medicine to represent that body in the International Medical Congress, to be held in Washington, Trelat, Verneuil, LeFort, Charpentier, Leon Laffe, Valin and Dujardin-Beaumetz.

---

During the month of November there were 342 deaths from Cholera Asiatica in Buenos Ayres and 145 deaths from cholera morbus. The population of Buenos Ayres is 400,000.

---

WILLARD ASYLUM for the chronic insane has established a training school for nurses, following the example of the Buffalo State Asylum, which established the first school of that sort in America.

---

BOCKELMANN has recently published an article showing that opacity of the cornea in new-born infants is often due to the contact of decomposing amniotic fluid with the eye.

